# Quantitative Assessment of the Association of COX-2 (Cyclooxygenase-2) Immunoexpression with Prognosis in Human Osteosarcoma: A Meta-Analysis

**DOI:** 10.1371/journal.pone.0082907

**Published:** 2013-12-16

**Authors:** Zhe Wang, Maolin He, Zengming Xiao, Hao Wu, Yang Wu

**Affiliations:** Division of Spine and Osteopathy Surgery, The First Affiliated Hospital of Guangxi Medical University, Nanning, Guangxi, China; Faculté de médecine de Nantes, France

## Abstract

**Background:**

Numerous studies examining the relationship between Cyclooxygenase-2 (COX-2) immunoexpression and clinical outcome in osteosarcoma patients have yielded inconclusive results.

**Methods:**

We accordingly conducted a meta-analysis of 9 studies (442 patients) that evaluated the correlation between COX-2 immunoexpression and clinical prognosis (death). Pooled odds ratios (OR) and risk ratios (RR) with 95% confidence intervals (95% CI) were calculated using the random-effects or fixed-effects model.

**Results:**

Meta–analysis showed no significant association between COX-2 positivity and age, gender, tumor location, histology, stage, metastasis or 90% necrosis. Conversely, COX-2 immunoexpression was associated with overall survival rate (RR=2.12; 95% CI: 1.10–3.74; P=0.009) and disease-free survival rate (RR=1.63; 95% CI: 1.17–2.28; P=0.004) at 2 years. Sensitivity analysis performed by omitting low quality studies showed that the pooled results were stable.

**Conclusions:**

COX-2 positivity was associated with a lower 2-year overall survival rate and disease-free survival rate. COX-2 expression change is an independent prognostic factor in patients with osteosarcoma.

## Introduction

Osteosarcoma is a life-threatening malignancy that often occurs in teenagers [1,2]. Its etiology is still unknown, but its genesis and progression may be regulated by genetic factors [3]. The administration of multiple chemotherapeutic agents before definitive resection of the primary tumor is a significant advance in treatment of osteosarcoma [4]. Nevertheless, multi-drug resistance and poor clinical outcome are problems encountered by about 50% of osteosarcoma patients [5]. The 5-year overall relapse-free survival rate is about 65% [6–8]. Therefore, a better understanding into its basic biology is urgently needed to identify its prognostic markers and therapeutic targets [9,10]. The mechanism of prognosis in osteosarcoma patients is still not fully understood. In recent years, several common genes have been identified to be in association with prognosis in human osteosarcoma. An important one is Cyclooxygenase (COX).

COX, also known as prostaglandin-endoperoxide synthase (PTGS), is the key enzyme in prostaglandin biosynthesis, and acts as both a dioxygenase and a peroxidase. COX has two isozymes: the constitutive COX-1 and the inducible COX-2, which differ in expression regulations and tissue distributions. This gene encodes the inducible isozyme. It is regulated by specific stimulatory events, suggesting that it is responsible for the prostanoid biosynthesis involved in inflammation and mitogenesis. Furthermore, COX-2 immunoexpression is associated with the prognosis of many human diseases, such as colorectal cancer [11], breast cancer [12], and clear cell renal cell carcinoma [13].

Numerous studies have reported the clinical significance of COX-2 overexpression in prognosis of osteosarcoma, but the results are inconclusive, partially because the effect of COX-2 immunoexpression on osteosarcoma outcomes is probably low and the sample size in each of published studies is relatively small. Therefore, we performed a meta-analysis of the published studies to estimate the association more accurately.

## Materials and Methods

### Publication search

This study was performed according to the proposal of Preferred Reporting Items for Systematic Reviews and Meta-Analyses (PRISMA) [14,15]. Databases PubMed (http://www.ncbi.nlm.nih.gov/pubmed/) and Embase (http://www.embase.com/) updated until July 2013 were searched electronically for all publications on the association of COX-2 expression with osteosarcoma outcomes. The search strategy was (‘osteosarcoma’ or ‘osteogenic sarcoma’) and (‘COX-2’ or ‘PTGS2’). Investigators were contacted and asked to supply additional data when relevant key information was missing.

### Inclusion criteria

No language or country restrictions were applied. All eligible studies were retrieved, and their bibliographies were checked for other relevant publications. Reviews and bibliographies of other relevant studies identified were searched by hand to find additional eligible studies. The inclusion criteria were as follows: (a) studies examining the relation between COX-2 expression and clinical outcome (death), (b) studies measuring COX-2 with immunohistochemistry (IHC) at protein level or reverse transcription-PCR (RT-PCR) for identifying gene changes, (c) cases were medically confirmed as osteosarcoma, (d) reported outcome measures with Kaplan–Meier curves or 2-year survival rate, and (e) case–control and cohort studies.

Whenever studies pertained to overlapped patients, only the largest-size study was retained to avoid duplication of information.

### Definition and standardization

For studies using IHC, prespecified rules were used to standardize, as much as possible, the definitions of a positive test for studies that used different cutoff thresholds. In this study, COX-2 protein positivity was defined as nuclear cell stain in more than 10% of the tumor cells, a definition followed by most studies. When different definitions were used, the cutoff to the 25% level or 60% level was accepted.

“Response to chemotherapy” was defined as the percentage of histologic necrosis of tumor cells in specimens obtained after chemotherapy. A cutoff of 90% necrosis was used to separate responders from nonresponders.

The clinical outcome of interest was mortality. Clinical outcomes were standardized to include a 24-month follow-up across all studies to avoid large time differentiation. All studies had at least 24 months of follow-up.

### Data extraction

Two investigators (ZW and MLH) extracted data from eligible studies independently, discussed discrepancies and reached consensus for all items. Data about the characteristics of studies and patients, measurements, and results were extracted. For each study, name(s) of author(s), journal and year of publication, country of origin, years of patient enrollment, number of patients analyzed, stage and grade of osteosarcoma, demographics, chemotherapy and surgery used, timing of COX-2 assessment (pre- or post-chemotherapy), type of COX-2 measurement, antibodies used for IHC, and definition(s) of COX-2 positivity were recorded. Data about the main outcomes were entered in 2×2 tables showing whether death occurred within 24 months depending on COX-2 status.

### Quality assessment

The methodological quality of each included case–control and cohort study was assessed on basis of Newcastle–Ottawa scale (NOS) [16]. A star system of NOS (0–9 stars) has been developed for the evaluation. The highest value is 9 stars (Table 1). Studies with 6 or more stars are rated as high quality.

**Table 1:** Methodological quality of studies included in the final analysis based on the Newcastle–Ottawa scale for assessing the quality of cohort studies.

Study(year)	Representativeness of the exposed cohort	Selection of the non-exposed cohort	Ascertain-ment of exposure	Outcome of interest was not present at start of study	Based on the design or analysis	Assessment of outcome	Follow-up long enough for outcomes to occur	Adequacy of follow-up of cohorts	Total score
David S. Dickens(2003)	1	1	1	1	2	1	1	1	9
Jiqing Li(2004)	1	1	1	1	1	0	1	1	7
Youqiao Liao(2007)	1	1	1	1	1	1	1	1	8
Yanhua Geng(2008)	1	1	1	1	1	1	1	1	8
Nidra I. Rodriguez(2008)	1	1	1	1	2	1	1	1	9
Xianbi Wang(2008)	1	1	1	0	1	0	0	1	5
Hiroshi Urakawa(2009)	1	1	1	1	2	1	1	1	9
I. V. Boulytcheva(2010)	1	1	1	0	1	0	0	1	5
Yong Chen(2012)	1	1	0	1	1	1	1	1	7

### Statistical analysis

Odds ratio (OR) was used to measure the relationship between COX-2 immunoexpression and clinical parameters. Data on the predictive ability of COX-2 for 24-month clinical outcomes were combined across studies in a similar way as random-effects estimates were used for synthesis of risk ratios (RR) for disease progression [17]. RR shows the 2-year mortality rate in the group with COX-2 overexpression or COX-2 gene alteration divided by the 2-year mortality rate in the group without COX-2 expression or COX-2 gene alteration. Between-study heterogeneity in RR was assessed with the Q statistic [17]. Fixed-effects models presume that differences between the results of the combined studies are due entirely to chance, while random-effects models allow for the possibility that results differ genuinely between studies. In the presence of between-study heterogeneity, random-effects models provide wider confidence intervals (CI) [18]. Therefore, random-effects estimates are generally presented in this study, unless stated otherwise.

Sensitivity analysis examines the effect of limiting the evaluations of high quality studies (with 6 or more stars). If the results do not change much when the articles are excluded, the sensitivity is low and the result is more robust and credible. On the contrary, if the results change much when the articles are excluded, the sensitivity is high and the result is less robust and credible.

Funnel plots were created for assessment of possible publication biases. Analyses were conducted on SPSS 16.0 and Review Manager 5.0.

## Results

### Characteristics of the studies

**Figure 1:**
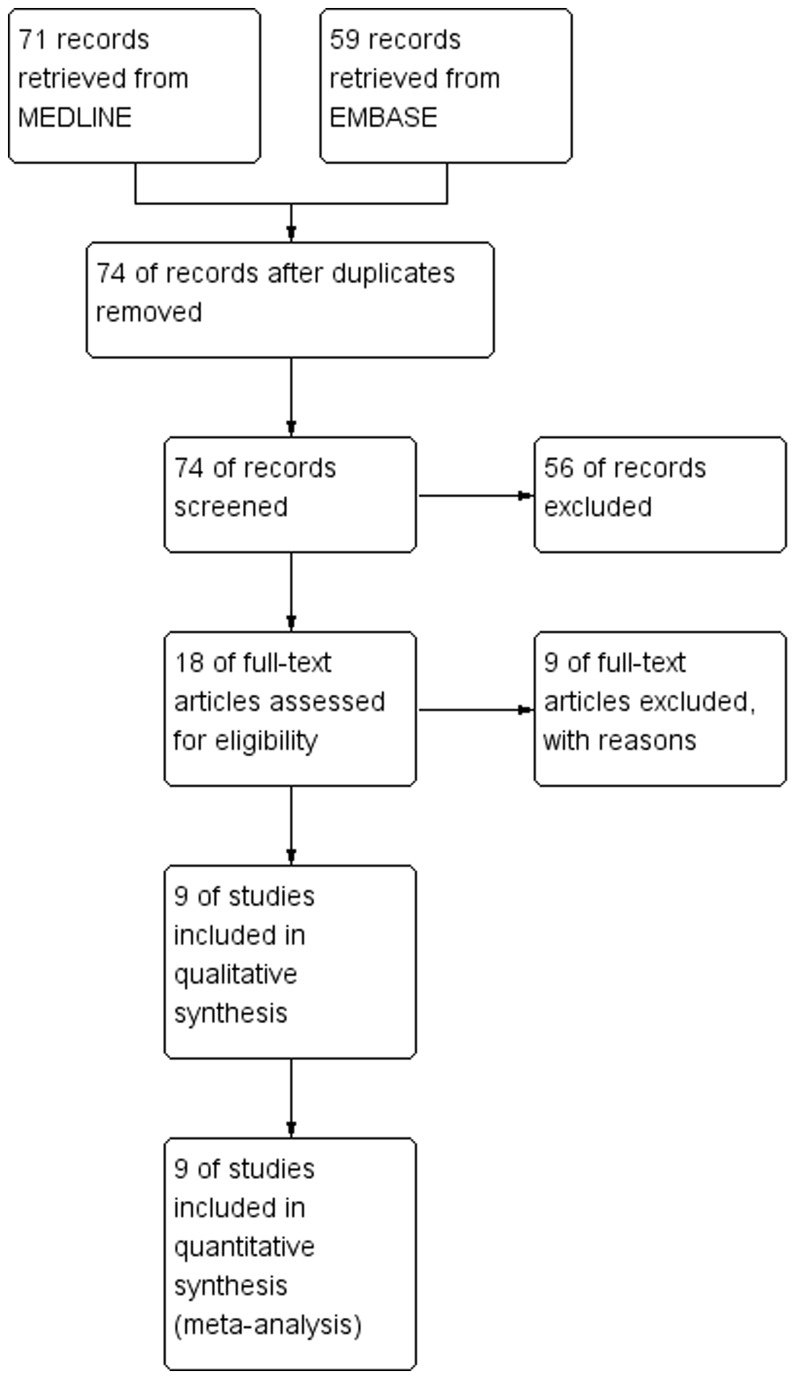
The process flow diagram describes how we filtered the data we retrieved.

We initially identified 18 studies evaluating the role of COX-2 status in osteosarcoma patients. Nine of them were excluded: 2 were reviews, 6 lacked some informative clinical data, and one overlapped with another study (Figure 1). In all, 9 independent eligible studies [19–27], which had data on 2-year survival rate and enrolled a total of 442 patients, were included in the quantitative synthesis.

Characteristics of the 9 studies are presented in Table 2. Among them, 6[19,21,23,25–27] were published in English and 3[20,22,24] in Chinese; 6 studies[20–22,24,25,27] were performed in Asians (Chinese and Japanese) and 3 studies[19,23,26] in Caucasians (Americans and Russians). The mean or median age of patients in each study ranged from 11.6 to 21 years; these populations were young. IHC was used to determine COX-2 status in all studies. COX-2 positivity was defined as more than 25% cutoff in 2 studies [19,20], as more than 60% cutoff in 1 study [21] and as more than 10% cutoff in 6 studies[22–27]. The antibodies used in these studies were not the same. Seven studies [19–25] provided data on overall survival rate (OS), while 5 studies [19,23,25–27] on disease-free survival rate (DFS). The overall quality of the included studies was adequate, with a mean value of 7.4 stars.

**Table 2:** Characteristics of Eligible Studies.

Ref.	Study (year)	Country	ethnicity	Patient(M/F)	Mean age	Method	Antibody source	COX-2 cutoff	Survival analysis	Quality score
[19]	David S. Dickens(2003)	America	Caucasians	45(24/21)	11.6	IHC	BioGenex	>25%	OS&DFS	9
[20]	Jiqing Li(2004)	China	Asian	50(28/22)	NR	IHC	NR	>25%	OS	7
[21]	Youqiao Liao(2007)	China	Asian	57(NR)	21	IHC	NR	>60%	OS	8
[22]	Yanhua Geng(2008)	China	Asian	59(20/39)	19.3	IHC	Maixin_Bio	>10%	OS	8
[23]	Nidra I. Rodriguez(2008)	America	Caucasians	36(NR)	NR	IHC	Santa Cruz	>10%	OS&DFS	9
[24]	Xianbi Wang(2008)	China	Asian	60(24/36)	17.3	IHC	Santa Cruz	>10%	OS	5
[25]	Hiroshi Urakawa(2009)	Japan	Asian	51(33/18)	15	IHC	Santa Cruz	>10%	OS&DFS	9
[26]	I. V. Boulytcheva(2010)	Russian	Caucasians	40(19/21)	NR	IHC	Thermo Scientifi	>10%	DFS	5
[27]	Yong Chen(2012)	China	Asian	49(28/21)	18.5	IHC	Abcam	>10%	DFS	7

NOTE. Antibodies, antibodies used for detection of COX-2 with IHC.

Abbreviations: NR, not reported; IHC, immunohistochemistry; OS, overall survival rate; DFS, disease-free survival rate.

### Data synthesis: association of COX-2 positivity with clinical parameters

**Figure 2:**
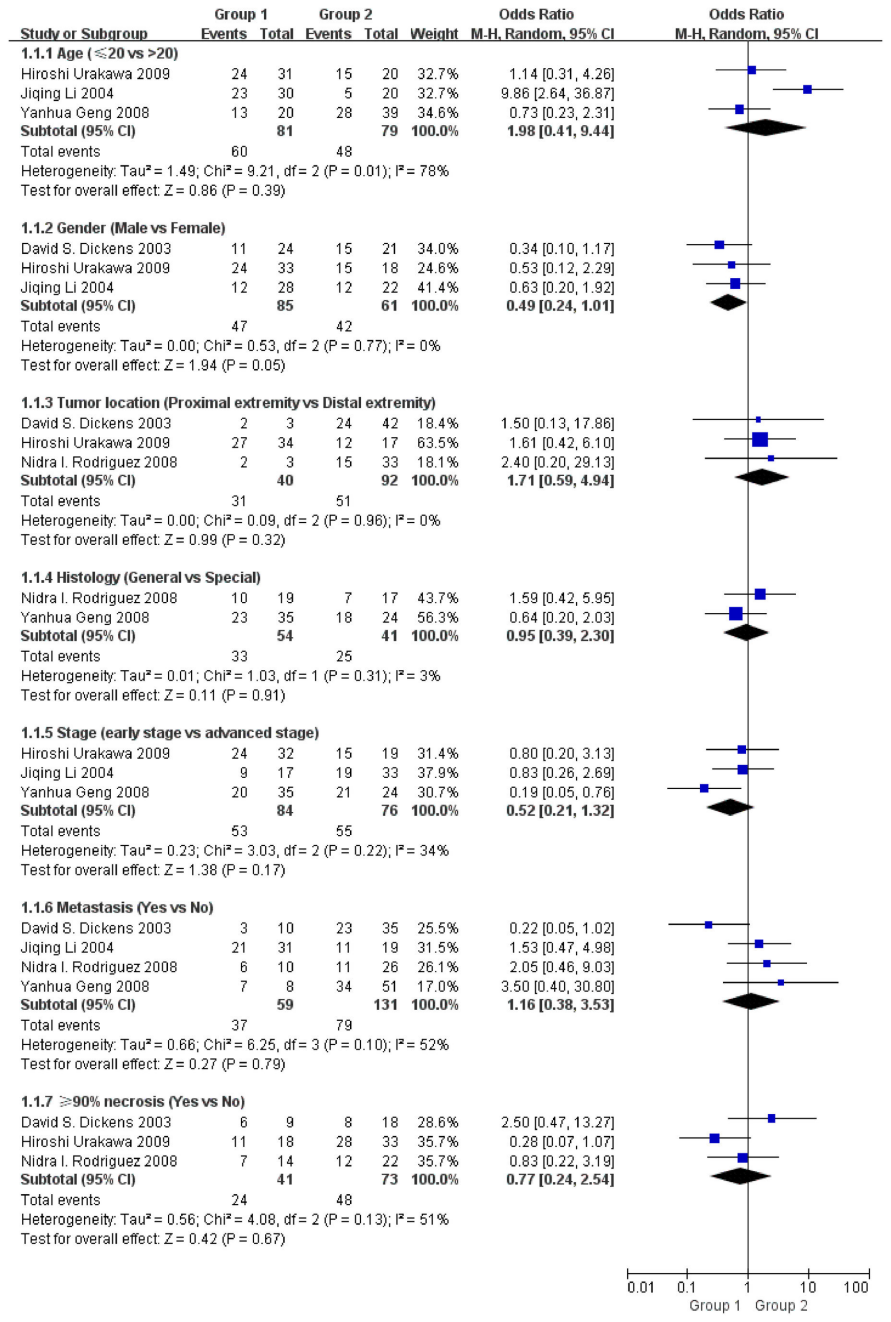
Funnel plot of the association of COX-2 positivity with clinical parameters.

Meta-analysis was performed on studies assessing the association between COX-2 positivity and age, gender, tumor location, histology, stage, metastasis or 90% necrosis. The pooled ORs were 1.98 (95% CI: 0.41–9.44, Z= 0.86, P= 0.39), 0.49 (95% CI: 0.24–1.01, Z= 1.94, P= 0.05), 1.71 (95% CI: 0.59–4.94, Z= 0.99, P= 0.32), 0.95 (95% CI: 0.39–2.30, Z= 0.11, P= 0.91), 0.52 (95% CI: 0.21–1.32, Z= 1.38, P= 0.17), 1.16 (95% CI: 0.38–3.53, Z= 0.27, P= 0.79) and 0.77 (95% CI: 0.24–2.54, Z = 0.42, P = 0.67) respectively (Figure 2). There was no significant association between COX-2 positivity and any of the above parameters.

### Data synthesis: overall survival rate (OS) at 2 years

**Figure 3:**
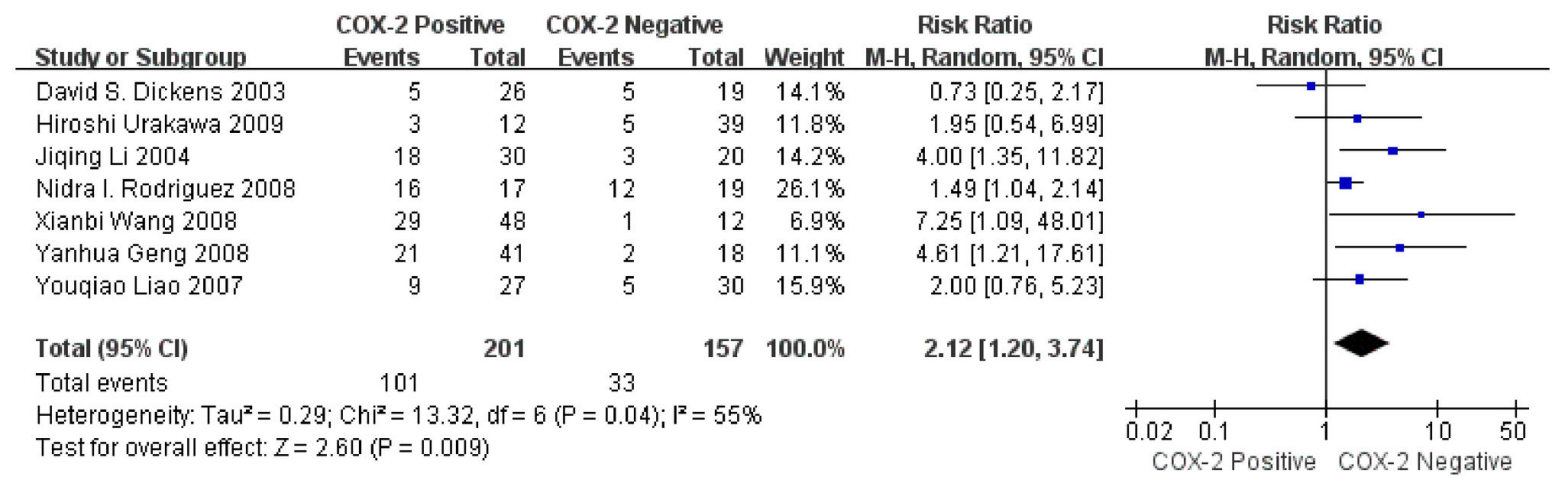
Funnel plot of the association between COX-2 positivity and overall survival rate at 2 years.

Seven studies assessed the association of COX-2 immunoexpression with 2-year OS in human osteosarcoma. The pooled RR was 2.12 (95% CI: 1.10–3.74; Z= 2.60; P= 0.009) (Figure 3) with heterogeneity (I^2^= 55%, P= 0.04). COX-2 positivity was associated with a low 2-year OS regarding the risk of death at 2 years.

**Figure 4:**
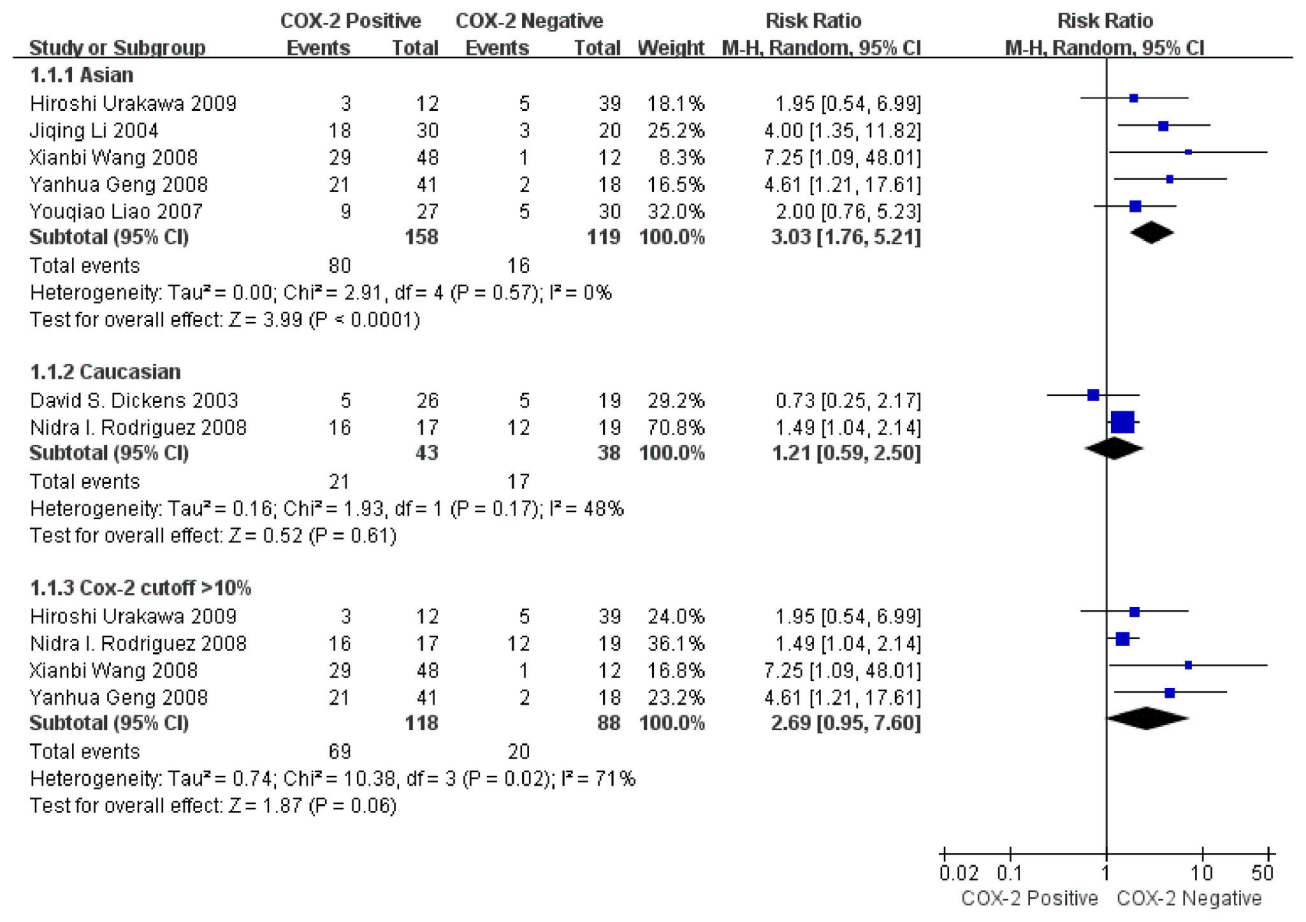
Funnel plot of the association between COX-2 positivity and overall survival rate at 2 years by subgroup analysis.

To explain the heterogeneity in OS, subgroup analysis was performed depending on ethnicity and definition of COX-2 positivity. A significant relationship between COX-2 immunoexpression and OS was found in Asians (RR=3.03, 95% CI: 1.76–5.21, Z= 3.99, P< 0.0001) without heterogeneity (I^2^= 0%, P= 0.57) (Figure 4), but not in Caucasians (RR= 1.21, 95% CI: 0.59–2.50, Z= 0.52, P= 0.61) without heterogeneity (I^2^= 48%, P= 0.17) (Figure 4). When COX-2 positivity was defined as a percentage, heterogeneity existed (I^2^= 71%, P= 0.02). It indicated that the difference of patient ethnicity contributed to the heterogeneity in the results.

**Figure 5:**
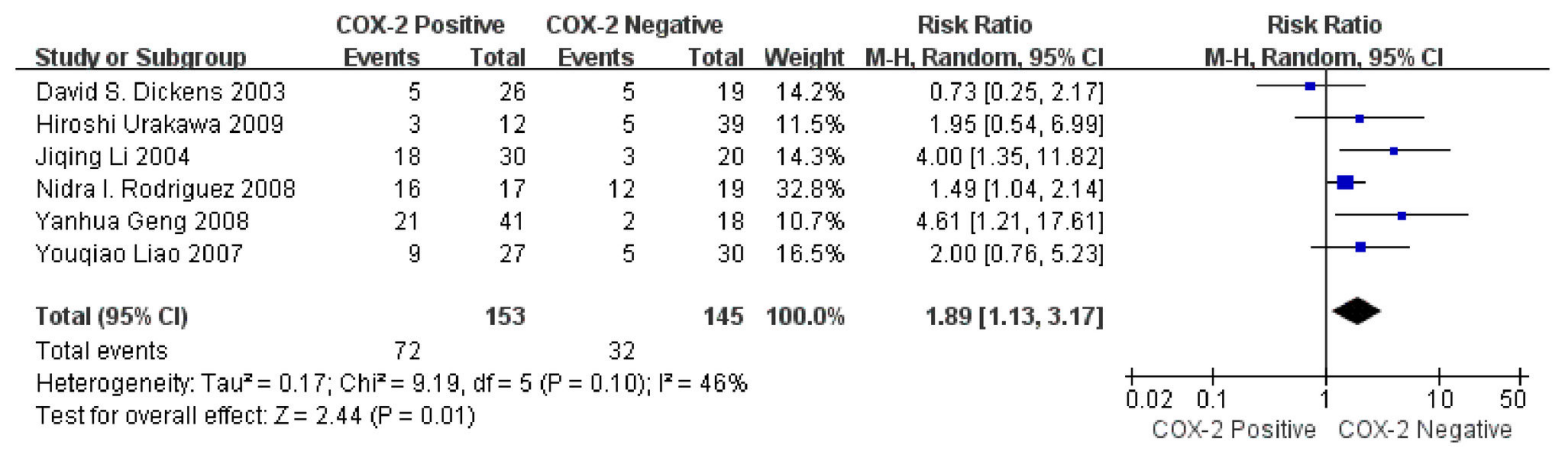
Funnel plot of the association between COX-2 positivity and overall survival rate at 2 years by Sensitivity analysis.

Sensitivity analysis was performed on six studies. The combined RR was 1.89 (95% CI: 1.13–3.17; Z= 2.44; P= 0.01) (Figure 5) without heterogeneity (I^2^= 46%, P= 0.10), indicating that the sensitivity is low and the result is more robust and credible.

### Data synthesis: disease-free survival rate (DFS) at 2 years

**Figure 6:**
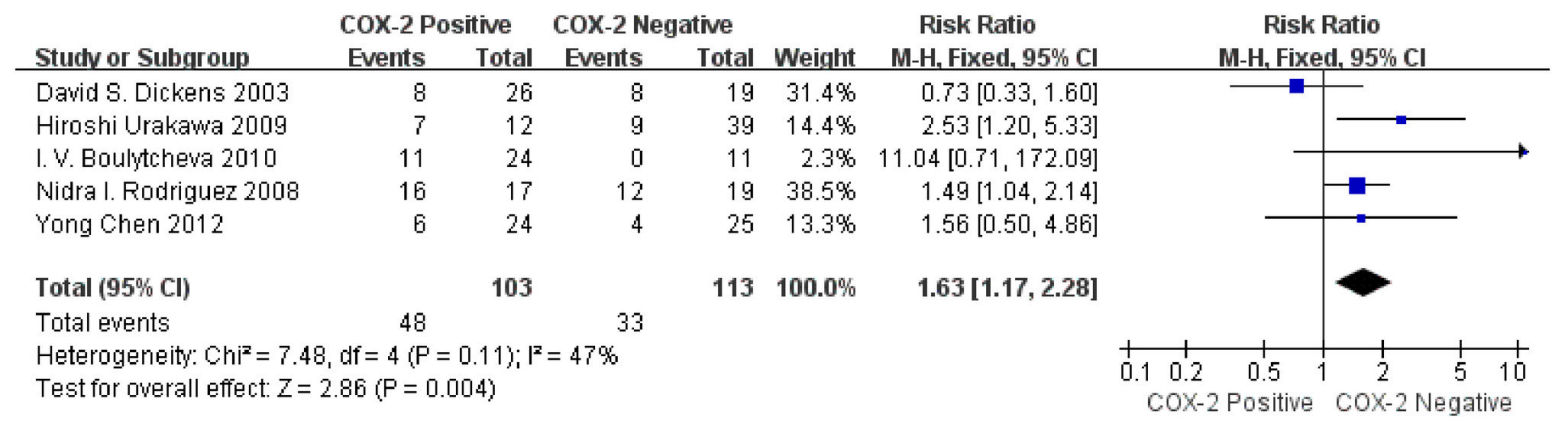
Funnel plot of the association between COX-2 positivity and disease-free survival rate at 2 years.

Meta-analysis was performed on five studies assessing the association of COX-2 immunoexpression with 2-year DFS in human osteosarcoma. The combined RR was 1.63 (95% CI: 1.17–2.28; Z= 2.86; P= 0.004) (Figure 6) without heterogeneity (I^2^= 47%, P= 0.11). COX-2 positivity was associated with a low 2-year DFS.

**Figure 7:**
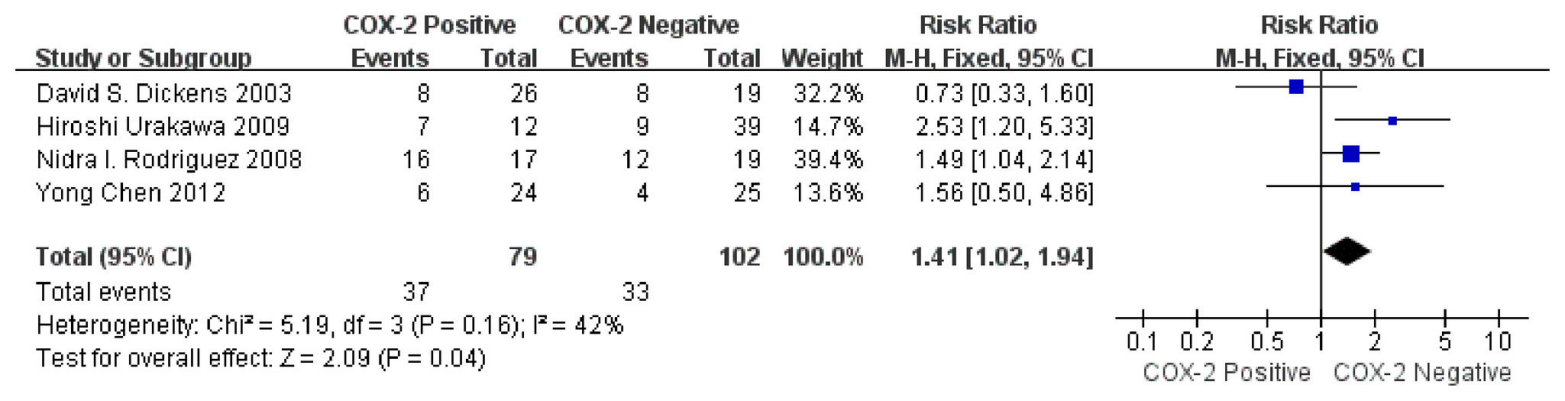
Funnel plot of the association between COX-2 positivity and disease-free survival rate at 2 years by Sensitivity analysis.

Sensitivity analysis was performed on four studies. The pooled RR was 1.41 (95% CI: 1.02–1.94; Z= 2.09; P= 0.04) (Figure 7) without heterogeneity (I^2^= 42%, P= 0.16), indicating that the sensitivity is low and the result is more robust and credible. These studies indicated that COX-2 immunoexpression was related to prognosis of osteosarcoma.

### Publication bias

Because the number of the included studies was comparatively small, we did not draw funnel plot to demonstrate publication bias.

## Discussion

### Summary of main results

Osteosarcoma is a very heterogenous disease entity and multiple factors affect its prognosis [2]. However, the molecular biomarkers for osteosarcoma are not well known, so we continue to carry out much research in the field. Whether COX-2 gene is a prognostic marker in osteosarcoma patients has been studied extensively, but the conclusions are inconsistent. This meta-analysis was carried out by critically reviewing 9 individual case–control studies on the association of COX-2 gene with prognosis in human osteosarcoma. Subgroup analyses were mainly done depending on ethnicity and definition of COX-2 positivity. Heterogeneity analysis and sensitivity analysis were also critically performed to ensure the epidemiological credibility of this meta-analysis. Through statistical study of 2-year survival rate, the following two basic conclusions are reached: (1) there is no significant association between COX-2 positivity and age, gender, tumor location, histology, stage, metastasis or 90% necrosis; (2) COX-2 positivity is associated with low 2-year OS and DFS.

### Summary of relevant literatures

The occurrence, development, invasion and metastasis of a malignant tumor are a process affected by multiple factors. The various biological functions of COX-2 are closely related to biological characteristics of malignant tumor. An increasing number of studies are revealing the relationship between them.

In the recent decade, meta-analyses show the significant association between many genes (e.g. TP53 [28], P-glycoprotein [29] and Ezrin [30]) and prognosis in osteosarcoma patients. 

In recent years, mounting evidence by meta-analysis also shows that COX-2 expression is associated with prognosis of various diseases, particularly cancer. Higher COX-2 expression may be an independent risk factor for low OS in patients with ovarian cancer [31]. COX-2 expression could be useful in distinguishing stage I non-small cell lung cancer (NSCLC) from those with worse prognosis [32]. COX-2 may play an important role in the progress of prostate cancer (PC), as its overexpression correlates with T3-T4 stages of PC. COX-2 is a potential therapy target for PC and may work as a prognostic factor for PC patients [33]. COX-2 overexpression may be an unfavorable prognostic and a chemoradiation resistance predictive factor for cervical cancer [34]. Moreover, COX-2 may play an important role in the progress of oesophageal squamous cell carcinoma (ESCC), as its overexpression correlates not only with the invasion depth and TNM stages, but also with the reduced OS. COX-2 is a potential therapy target for ESCC and may work as a prognostic factor for ESCC patients [35]. 

### Comparison with other relevant work

Recently, many meta-analyses are performed to investigate the association between many genes (e.g. VEGF [36], HER-2[37], TP53 [28], P-glycoprotein [29] and Ezrin [30]) and prognosis in osteosarcoma patients. Significant association was found in TP53 [28], P-glycoprotein [29] and Ezrin [30], but not in VEGF[36] or HER-2[37]. 

When the present manuscript was being written, a meta-analysis about COX-2 immunoexpression on the prognosis of osteosarcoma patients was published [38]. There are some shortcomings which may have a negative effect on the reliability of the final results. Firstly, the 14 eligible studies are comprised of 10 papers from China and 4 papers from other countries. Bias was not fully considered by the authors. Secondly, some low quality literatures were included in their meta-analysis. What’s worse, the authors did not evaluate the quality of the literatures and they only pooled all the data from eligible studies, which may substantially affect the final results. Finally, in analysis of prognosis composition, the authors did not extract the relevant data from the majority of eligible studies. They simply extracted and pooled the data from 4 literatures, and acquired a negative result that high COX-2 expression tended to be associated with a poor 3-year survival (the difference was not significant). The reliability of the result that high COX-2 expression might have an unfavorable prognostic effect on osteosarcoma is questionable. Therefore, it is necessary to update by meta-analysis to comprehensively investigate the relationship between COX-2 immunoexpression and prognosis of osteosarcoma patients. In our study, however, COX-2 positivity was associated with a low 2-year OS and DFS. Our findings suggest that COX-2 expression change is an independent prognostic factor in patients with osteosarcoma.

In addition, previous meta-analyses did not pay attention to heterogeneity. Heterogeneity is a potential problem when interpreting the results of all meta-analyses, and finding the sources of heterogeneity is one key goal of meta-analysis. In the present meta-analysis, between-study heterogeneity was assessed by using two methods including the chi-square-based Q statistic for testing and the I^2^ statistic for quantification. The results show significant between-study heterogeneity in OS. To find the major sources of heterogeneity, subgroup meta-analyses were first performed depending on ethnicity and definition of COX-2 positivity. Heterogeneity was still significant in the definition of COX-2 positivity, while it was removed in ethnicity, indicating that heterogeneity might result from the inconsistency of effects across those studies included from different populations.

### Strengths of the meta-analysis

There are some shortcomings in the former study. It is necessary to update by meta-analysis to comprehensively investigate the relationship between COX-2 immunoexpression and prognosis of osteosarcoma patients. To this end, we carried out this work. This work was performed according to the proposal of Preferred Reporting Items for Systematic Reviews and Meta-Analyses (PRISMA). By statistical analysis of 2-year survival rate, this meta-analysis draws a meaningful conclusion that COX-2 positivity is associated with a low 2-year OS and DFS.

### Limitations

Several limitations of this meta-analysis are acknowledged. First, only published studies were included. Probably some relevant unpublished studies that meet the inclusion criteria are missed; therefore, publication bias may be present. We tried to identify all relevant data and retrieve additional unpublished information, but data missing was unavoidable. Typically, publication bias results in seeing stronger associations in small-size studies than in large-size studies. However, a stronger association of COX-2 positive status with 2-year mortality rate was reassuringly observed in large-size studies. Thus, the association was clearer in high-quality studies. Second, some variability in definitions of methods, measurements, and outcomes among all studies was unavoidable, despite the effort to standardize definitions. Third, the number of the included studies was not sufficiently large for a comprehensive analysis, but given that osteosarcoma is not very common on a population basis, the sample size of this investigation is one of the largest to date among studies targeting this malignancy. Fourth, the literatures included in our meta-analysis were published from 2003 to 2012. The articles published five years ago whose methods were applied to the therapy of osteosarcoma may differ from the nearest published articles, which may affect the overall survival. Fifth, only 3 of the 9 papers involve the Caucasian population. Some literatures suggest that osteosarcoma among different ethnicities may respond to similar treatment differently. Other ethnicities including mixed and Africans should be investigated in future studies. Sixth, with subgroup analysis of clinical parameters, only data from 2-3 papers were used for each subgroup. This could represent a skewed analysis of the results and some of these confounders may in fact be significant.

## Conclusions

Our findings suggest that COX-2 expression change is an independent prognostic factor in patients with osteosarcoma. But current studies are still controversial in some aspects. For better understanding the relationship between COX-2 expression and osteosarcoma outcomes, it is necessary to improve the experimental and detection methods, and to unify a quantitative standard. The mechanism of COX-2 expression in osteosarcoma patients is not clear yet. With further research, COX-2 might become another target of the treatment of osteosarcoma.

## Supporting Information

Checklist S1 **PRISMA checklist.**
(DOC)

## References

[1] Ottaviani G, Jaffe N (2009) The etiology of osteosarcoma. Cancer Treat Res 152: 15-32. doi:10.1007/978-1-4419-0284-9_2. PubMed: 20213384. 20213384

[2] Bertucci F, Araujo J, Giovannini M (2013) Pancreatic metastasis from osteosarcoma and Ewing sarcoma: literature review. Scand J Gastroenterol 48: 4-8. doi:10.3109/00365521.2012.711852. PubMed: 22861647. 22861647

[3] Fuchs B, Zhang K, Schabel A, Bolander ME, Sarkar G (2001) Identification of twenty-two candidate markers for human osteogenic sarcoma. Gene 278: 245-252. doi:10.1016/S0378-1119(01)00731-4. PubMed: 11707342. 11707342

[4] Rosen G, Murphy ML, Huvos AG, Gutierrez M, Marcove RC (1976) Chemotherapy, en bloc resection, and prosthetic bone replacement in the treatment of osteogenic sarcoma. Cancer 37: 1-11. doi:10.1002/1097-0142(197601)37:1. PubMed: 1082364. 1082364

[5] Scotlandi K, Serra M, Nicoletti G, Vaccari M, Manara MC et al. (1996) Multidrug resistance and malignancy in human osteosarcoma. Cancer Res 56: 2434-2439. PubMed: 8625324. 8625324

[6] Gorlick R (2009) Current concepts on the molecular biology of osteosarcoma. Cancer Treat Res 152: 467-478. doi:10.1007/978-1-4419-0284-9_27. PubMed: 20213409. 20213409

[7] Foster L, Dall GF, Reid R, Wallace WH, Porter DE (2007) Twentieth-century survival from osteosarcoma in childhood. Trends from 1933 to 2004. J Bone Joint Surg Br 89: 1234-1238. 17905964 10.1302/0301-620X.89B9.19255

[8] Hagleitner MM, de Bont ES, Te Loo DM (2012) Survival trends and long-term toxicity in pediatric patients with osteosarcoma. Sarcoma 2012: 636405. 23226967 10.1155/2012/636405 PMC3512330

[9] Gorlick R (2002) Osteosarcoma: clinical practice and the expanding role of biology. J Musculoskelet Neuronal Interact 2: 549-551. PubMed: 15758391. 15758391

[10] Durnali A, Alkis N, Cangur S, Yukruk FA, Inal A et al. (2013) Prognostic factors for teenage and adult patients with high-grade osteosarcoma: an analysis of 240 patients. Med Oncol 30: 624. doi:10.1007/s12032-013-0624-6. PubMed: 23749307. 23749307

[11] Yamauchi M, Lochhead P, Imamura Y, Kuchiba A, Liao X et al. (2013) Physical Activity, Tumor PTGS2 Expression, and Survival in Patients with Colorectal Cancer. Cancer Epidemiol Biomarkers Prev 22: 1142-1152. doi:10.1158/1055-9965.EPI-13-0108. 23629521 PMC3681847

[12] Sproviero D, Julien S, Burford B, Taylor-Papadimitriou J, Burchell JM (2012) Cyclooxygenase-2 induces the expression of the alpha 2,3 sialyltransferase-3 (ST3Gal-I) in breast cancer. J Biol Chem. 10.1074/jbc.M112.427827 PMC3531762 23275522

[13] Lee JW, Park JH, Suh JH, Nam KH, Choe JY et al. (2012) Cyclooxygenase-2 expression and its prognostic significance in clear cell renal cell carcinoma. Korean J Pathol 46: 237-245. 23110009 10.4132/KoreanJPathol.2012.46.3.237 PMC3479766

[14] Moher D, Altman DG, Liberati A, Tetzlaff J (2011) PRISMA statement. Epidemiology 22: 128; author reply: 21150360 10.1097/EDE.0b013e3181fe7825

[15] Moher D, Liberati A, Tetzlaff J, Altman DG (2009) Preferred reporting items for systematic reviews and meta-analyses: the PRISMA statement. PLOS Med 6: e1000097. 19621072 10.1371/journal.pmed.1000097 PMC2707599

[16] Stang A (2010) Critical evaluation of the Newcastle-Ottawa scale for the assessment of the quality of nonrandomized studies in meta-analyses. Eur J Epidemiol 25: 603-605. doi:10.1007/s10654-010-9491-z. PubMed: 20652370. 20652370

[17] Petitti DB (2000) Meta-analysis, decision analysis, and cost-effectiveness analysis : methods for quantitative synthesis in medicine. New York: Oxford University Press. 306 p.

[18] Lau J, Ioannidis JP, Schmid CH (1997) Quantitative synthesis in systematic reviews. Ann Intern Med 127: 820-826. doi:10.7326/0003-4819-127-9-199711010-00008. PubMed: 9382404. 9382404

[19] Dickens DS, Kozielski R, Leavey PJ, Timmons C, Cripe TP (2003) Cyclooxygenase-2 expression does not correlate with outcome in osteosarcoma or rhabdomyosarcoma. J Pediatr Hematol Oncol 25: 282-285. doi:10.1097/00043426-200304000-00003. PubMed: 12679640. 12679640

[20] Li JQ, Wang Z, Guo Z, Guo F, Yuan Z et al. (2004) Relationship between the protein expressions of cyclooxygenase-2 and matrix metalloproteinase-7 in high-risk osteosarcoma and the related factor of prognosis such as age, gender, onset time. Zhongguo Linchuang Kangfu 8: 1488-1489.

[21] Liao Y, Li F, Hu X (2007) Expression and clinical significance of OPN and COX-2 in osteosarcoma. The Chinese-German Journal of Clinical Oncology 6: 378-382.

[22] Yan-hua G, hang-xing W, Pe-ihui C (2008) Expressions of Cox 2 and HIF-1A and their relationship with clinicopathologic characteristics of osteosarcoma. Tumor 28: 427-430.

[23] Rodriguez NI, Hoots WK, Koshkina NV, Morales-Arias JA, Arndt CA et al. (2008) COX-2 expression correlates with survival in patients with osteosarcoma lung metastases. J Pediatr Hematol Oncol 30: 507-512. doi:10.1097/MPH.0b013e31816e238c. PubMed: 18797196. 18797196 PMC2771732

[24] Xianbin W, Liguo S, Xiaoju Z (2008) Expression of COX-2 Protein and Prognosis Significance in Human Osteosarcoma. Chinese Journal Clinical Practical Medicine 9: 19-20.

[25] Urakawa H, Nishida Y, Naruse T, Nakashima H, Ishiguro N (2009) Cyclooxygenase-2 overexpression predicts poor survival in patients with high-grade extremity osteosarcoma: a pilot study. Clin Orthop Relat Res 467: 2932-2938. doi:10.1007/s11999-009-0814-x. PubMed: 19326179. 19326179 PMC2758970

[26] Boulytcheva IV, Soloviev YN, Kushlinskii NE, Mahson AN (2010) Expression of molecular markers in the tumor and survival prognosis in osteosarcoma. Bull Exp Biol Med 150: 237-242. doi:10.1007/s10517-010-1114-x. PubMed: 21240382. 21240382

[27] Chen Y, Yang Y, Yuan Z, Wang C, Shi Y (2012) Predicting chemosensitivity in osteosarcoma prior to chemotherapy: An investigational study of biomarkers with immunohistochemistry. Oncol Lett 3: 1011-1016. PubMed: 22783382. 22783382 10.3892/ol.2012.604 PMC3389645

[28] Pakos EE, Kyzas PA, Ioannidis JP (2004) Prognostic significance of TP53 tumor suppressor gene expression and mutations in human osteosarcoma: a meta-analysis. Clin Cancer Res 10: 6208-6214. doi:10.1158/1078-0432.CCR-04-0246. PubMed: 15448009. 15448009

[29] Pakos EE, Ioannidis JP (2003) The association of P-glycoprotein with response to chemotherapy and clinical outcome in patients with osteosarcoma. A meta-analysis. Cancer 98: 581-589. doi:10.1002/cncr.11546. PubMed: 12879476. 12879476

[30] Wang Z, He ML, Zhao JM, Qing HH, Wu Y (2013) Meta-analysis of Associations of the Ezrin Gene with Human Osteosarcoma Response to Chemotherapy and Prognosis. Asian Pac J Cancer Prev 14: 2753-2758. doi:10.7314/APJCP.2013.14.5.2753. PubMed: 23803027. 23803027

[31] Lee JY, Myung SK, Song YS (2013) Prognostic role of cyclooxygenase-2 in epithelial ovarian cancer: a meta-analysis of observational studies. Gynecol Oncol 129: 613-619. doi:10.1016/j.ygyno.2013.02.011. PubMed: 23422504. 23422504

[32] Zhan P, Qian Q, Yu LK (2013) Prognostic value of COX-2 expression in patients with non-small cell lung cancer: a systematic review and meta-analysis. J Thorac Dis 5: 40-47. 23372950 10.3978/j.issn.2072-1439.2013.01.02 PMC3547998

[33] Shao N, Feng N, Wang Y, Mi Y, Li T et al. (2012) Systematic review and meta-analysis of COX-2 expression and polymorphisms in prostate cancer. Mol Biol Rep 39: 10997-11004. doi:10.1007/s11033-012-2001-5. PubMed: 23053989. 23053989

[34] Huang M, Chen Q, Xiao J, Liu C, Zhao X (2013) Prognostic significance of cyclooxygenase-2 in cervical cancer: a meta-analysis. Int J Cancer 132: 363-373. doi:10.1002/ijc.27686. PubMed: 22729746. 22729746

[35] Li L, Zhao J, Wu Z, Wang G, Chen G (2009) Meta-analysis: clinicopathological and prognostic significance of cyclooxygenase-2 expression on oesophageal squamous cell carcinoma. Aliment Pharmacol Ther 30: 589-596. doi:10.1111/j.1365-2036.2009.04069.x. PubMed: 19549265. 19549265

[36] Qu JT, Wang M, He HL, Tang Y, Ye XJ (2012) The prognostic value of elevated vascular endothelial growth factor in patients with osteosarcoma: a meta-analysis and systemic review. J Cancer Res Clin Oncol 138: 819-825. doi:10.1007/s00432-012-1149-7. PubMed: 22274866. 22274866 PMC11824587

[37] Li YG, Geng X (2010) A meta-analysis on the association of HER-2 overexpression with prognosis in human osteosarcoma. Eur J Cancer Care (Engl) 19: 313-316. doi:10.1111/j.1365-2354.2008.00970.x. 19709164

[38] Jiao G, Ren T, Lu Q, Sun Y, Lou Z et al. (2013) Prognostic significance of cyclooxygenase-2 in osteosarcoma: a meta-analysis. Tumour Biol 34: 2489–2495. PubMed: 23857285. 23857285 10.1007/s13277-013-0998-2

